# Self-Recognition Sensitizes Mouse and Human Regulatory T Cells to Low-Dose CD28 Superagonist Stimulation

**DOI:** 10.3389/fimmu.2017.01985

**Published:** 2018-01-30

**Authors:** Daniela Langenhorst, Paula Tabares, Tobias Gulde, Bryan R. Becklund, Susanne Berr, Charles D. Surh, Niklas Beyersdorf, Thomas Hünig

**Affiliations:** ^1^Institute for Virology and Immunobiology, University of Würzburg, Würzburg, Germany; ^2^Department of Immunology and Microbial Science, Scripps Research Institute, La Jolla, CA, United States; ^3^Division of Developmental Immunology, La Jolla Institute for Allergy and Immunology, La Jolla, CA, United States; ^4^Academy of Immunology and Microbiology, Institute for Basic Science, Pohang, South Korea; ^5^Department of Integrative Biosciences and Biotechnology, Pohang University of Science and Technology, Pohang, South Korea

**Keywords:** regulatory T cells, self-reactivity, autoimmunity, CD28 superagonists, TGN1412, TAB08, D665

## Abstract

In rodents, low doses of CD28-specific superagonistic monoclonal antibodies (CD28 superagonists, CD28SA) selectively activate regulatory T cells (Treg). This observation has recently been extended to humans, suggesting an option for the treatment of autoimmune and inflammatory diseases. However, a mechanistic explanation for this phenomenon is still lacking. Given that CD28SA amplify T cell receptor (TCR) signals, we tested the hypothesis that the weak tonic TCR signals received by conventional CD4^+^ T cells (Tconv) in the absence of cognate antigen require more CD28 signaling input for full activation than the stronger TCR signals received by self-reactive Treg. We report that *in vitro*, the response of mouse Treg and Tconv to CD28SA strongly depends on MHC class II expression by antigen-presenting cells. To separate the effect of tonic TCR signals from self-peptide recognition, we compared the response of wild-type Treg and Tconv to low and high CD28SA doses upon transfer into wild-type or H-2M knockout mice, which lack a self-peptide repertoire. We found that the superior response of Treg to low CD28SA doses was lost in the absence of self-peptide presentation. We also tested if potentially pathogenic autoreactive Tconv would benefit from self-recognition-induced sensitivity to CD28SA stimulation by transferring TCR transgenic OVA-specific Tconv into OVA-expressing mice and found that low-dose CD28SA application inhibited, rather than supported, their expansion, presumably due to the massive concomitant activation of Treg. Finally, we report that also in the *in vitro* response of human peripheral blood mononuclear cells to CD28SA, HLA II blockade interferes with the expansion of Treg by low-dose CD28SA stimulation. These results provide a rational basis for the further development of low-dose CD28SA therapy for the improvement of Treg activity.

## Introduction

CD28 superagonists (CD28SA) are a unique class of CD28-specific monoclonal antibodies (mAb) able to activate T cells without overt stimulation of the T cell receptor (TCR) (1, 2). A common feature of CD28SA specific for rat, mouse, and human CD28 is their lateral binding mode to the CD28 homodimer, which allows lattice formation, a feature likely to contribute to their strong agonistic properties (3, 4). In contrast, conventional CD28-specific mAb, which synergize with TCR ligation in T cell activation, bind monovalently at the ligand binding site (5), a feature which they share with the natural ligands CD80 and CD86 (6). While CD28SA activate T cells both *in vitro* and *in vivo* without TCR ligation by mAb or MHC molecules presenting cognate peptide antigens, this activation strictly depends on “tonic” TCR signals (7, 8) generated by cellular interactions (9) during the process known as MHC scanning, in which the TCR briefly docks onto MHC peptide complexes in a MHC class and allele-non-specific fashion and rapidly dissociates unless a cognate peptide is recognized (10).

This strict dependence of the T cell response to CD28SA on preactivation through cell–cell contacts in the tissue results in the inability of human circulating T cells to respond to the human CD28SA TGN1412 (now called TAB08), which contributed to the failure to predict the cytokine release syndrome triggered by this antibody during a first-in-human (FIH) trial in 2006 (11, 12). In the meantime, a method has been developed which resets human peripheral blood mononuclear cells (PBMC) to tissue-like status, allowing the *in vitro* analysis of the response to this potent T cell activating agent (9).

Using this cell-culture system, we have recently reported the response of human Tconv and regulatory T cells (Treg) to titrated concentrations of TAB08 (13). We found that *in vitro* stimulation with CD28SA concentrations equivalent to those reached during the failed FIH trial of 2006 results in maximum release of pro-inflammatory cytokines from CD4^+^ effector memory (CD4EM) T cells, accompanied by a strong expansion of Treg. Furthermore, reduction of the CD28SA concentration resulted in a complete loss of pro-inflammatory cytokine release at concentrations which still induced substantial Treg activation. These findings provided experimental support for the feasibility of a new FIH study, in which TAB08 was applied at doses ranging from 1/1,000 to 1/14 of the 2006 trial dose. While no adverse effects were observed and the pro-inflammatory cytokines in the circulation remained at baseline with these low doses of CD28SA, there was a time- and dose-dependent release of the Treg signature cytokine IL-10 into the blood stream (13).

These results confirmed for humans what had initially been observed in rodents, i.e., the particular sensitivity of Treg as compared to Tconv to CD28SA stimulation, a finding which had formed the basis of the translational development of the CD28SA TGN1412 for the treatment of autoimmune and inflammatory conditions. Thus, both in rats (14) and in mice (15), application of low CD28SA doses results in selective expansion of Treg, whereas both conventional and Treg cells are activated by high CD28SA doses. It is worth mentioning that even when high doses of CD28SA are applied to rodents, no toxic cytokine release syndrome is observed because the few CD4EM T cells present in clean laboratory rodents are effectively controlled by the powerful Treg response (15).

While the selectivity of low-dose CD28SA treatment for Treg activation opens a therapeutic window for the treatment of autoimmune and inflammatory diseases, it is, so far, mechanistically not understood. Here, we hypothesized that this effect is due to a stronger TCR input signal perceived by the self-reactive regulatory as opposed to the non-self-specific conventional CD4^+^ T cells which receive only the weak signal generated by MHC scanning, providing more substrate for signal amplification through the CD28 pathway. Indeed, *ex vivo* biochemical analysis of the TCR complex in mice has revealed a higher degree of TCRζ phosphorylation in Treg over Tconv, which was abolished by preventing MHC class II recognition through mAb blockade (16). We here show that indeed, the high sensitivity of murine and human Treg to CD28SA stimulation depends on MHC II recognition *in vitro* and that prevention of self-peptide recognition by genetic interference with MHC II peptide loading (17) similarly abrogates preferential Treg activation *in vivo*.

## Materials and Methods

### Peripheral Blood Mononuclear Cells

Human PBMC were prepared from healthy donors as a byproduct of platelet concentrates obtained with leukoreduction system chambers (Gambro Trima Accel aphaeresis apparatus, Pall Corporation) (18) and diluted in versene solution (0.7 mM EDTA in PBS).

### Mice

C57BL/6 (Harlan Winkelmann, Borchen, Germany), OT-I Thy1.1^+/−^, and OT-II Thy1.1^+/+^ mice were bred in the institute’s barrier facility. H-2M^−/−^ (17, 19), C57BL/6.CD11c-DOG (20), and MHC II^−/−^ mice (C57BL/6.129S2-H2^dlAb1-Ea^/J) (21) were generously provided by Wei-Ping Fung-Leung (LLC Janssen Research & Development, San Diego, CA, USA), Andreas Beilhack (Department of Internal Medicine II, University Hospital Würzburg, Würzburg, Germany), and Lars Nitschke (Division of Genetics, Department of Biology, University of Erlangen-Nürnberg, Erlangen, Germany), respectively.

### Cell Culture and Stimulation Assays (Human)

Cells were cultured in RPMI 1640 supplemented with l-glutamine (Gibco), non-essential amino acids (Gibco), HEPES (Applichem), β-mercaptoethanol (Gibco), sodium pyruvate (Gibco), penicillin/streptomycin, and 10% AB-positive heat-inactivated human serum (Sigma) (AB medium). PBMC were first cultured for 2 days at a high cell density (1 × 10^7^/ml) in AB medium to reset them to tissue-like conditions without changing their cellular composition, thereby allowing reactivity to TGN1412/TAB08 in the secondary cultures. For these, cells were harvested and cultured under standard conditions (1 × 10^6^/ml) in 48-well flat-bottom tissue culture plates in a final volume of 0.6 ml of supplemented RPMI/AB medium for 5 days in a humidified incubator at 37°C with 5% CO_2_. GMP-grade TAB08 was provided by TheraMAB GmbH. Pan HLA II-specific Tü39 antibodies were provided by Hans-Georg Rammensee from Tübingen, Germany. Isotype control IgG2a was from Abcam. Fab fragments were prepared using the Pierce Fab Preparation Kit (Thermo Scientific).

### Cell Proliferation Assay Using CFSE or Cell Trace Violet (CTV)-Labeled Cells

T cell proliferation was monitored by the stepwise dilution of fluorescence in CFSE- or CTV-labeled cells. To label with CFSE (Life technologies, Eugene, DR, USA) or CTV (Life technologies, Eugene, DR, USA), the cells were washed with PBS and incubated with 5 µM CFSE or CellTrace™ Violet for 5–20 min at RT. To remove excess dye, the cells were washed twice with RPMI medium.

### Cell Culture and Stimulation Assays (Mice)

Purified CD4^+^ cells from C57BL/6 mice were cultured for 4 days in U-bottomed 96-well plates (1 × 10^5^ cells/well) together with T cell-depleted splenocytes from C57BL/6 or MHC II ko mice (2.5 × 10^5^ cells/well) and various concentrations of CD28SA D665 (5) (manufactured by Exbio, Prague, Czech Republic), in the presence and absence of IL-2 (200 U/ml, Novartis, Basel, CH). To follow proliferation, cells were labeled with CFSE. Fold increase was calculated as: absolute cell number with × μg CD28SA/absolute cell number put into culture on day 0. Average cell division (acd) numbers were calculated as ∑[% of cells in generation (*n*) × *n*]/100 (*n* = number of generation).

### Antibodies and Flow Cytometry

The following anti-human antibodies were used: CD4-PE/Cy5, CD25-FITC, and Foxp3-Alexa647 from BioLegend (San Diego, CA, USA). Mouse cells were stained with fluorochrome-labeled mAbs to CD4-Pacific Blue, Brilliant Violet 605 and Alexa-700 (RM4-5), CD8-Pacific Blue (53-6.7), CD25-APC (7D4), Ki-67-PE (Ki-67), and Thy1.1-PercP (Ox-7) from BioLegend. Foxp3-APC (FJK-16s), dead cell marker Viability Dye eFluor™ 780, and staining reagents from eBioscience (San Diego, CA, USA) were used according to the manufacturer’s instructions. To analyze expression of surface proteins, the cells were stained with the appropriate antibodies for 20 min at 4°C, washed once with FACS buffer (PBS, 0.1% BSA, and 0.02% NaN_3_), and fixed with 2% paraformaldehyde. For intracellular staining of Foxp3 and Ki-67, the cells were first surface stained, permeabilized with Fix/Perm (eBioscience), and stained with Foxp3 and Ki-67 antibodies diluted in Perm/Wash (eBioscience). To calculate absolute Treg numbers, unlabeled microbeads (BD Biosciences, Franklin Lakes, NJ, USA) were added to the stained cells and the following formula was used: absolute Treg numbers = (beads used × Treg events)/beads measured. Acquisition was performed on a BD™ LSR II or FACSCalibur, and data were analyzed using FlowJo software (TreeStar Inc., Ashland, OR, USA).

### Cell Isolation, Labeling, and Cell Transfer (Mice)

Single-cell suspensions of lymph nodes were stained with a cocktail of biotin-labeled antibodies (BD Biosciences), followed by incubation with Streptavidin MicroBeads. CD4^+^ and CD8^+^ T cells were prepared by negative selection using the MACS separation system (Miltenyi Biotec, Bergisch Gladbach, Germany). Purified CD4^+^ T cells and CD8^+^ T cells of untreated C57BL/6, OT-II Thy1.1^+/+^, or OT-I Thy1.1^+/−^ mice were labeled with CFSE or CellTrace™ Violet, resuspended in PBS (cell numbers as indicated), and transferred i.v. 12–24 h before CD28SA stimulation. Per mouse 25–150 µg CD28SA was injected intraperitoneally. Mice were analyzed 3–4 days after cell transfer. Average cell division (acd) numbers of transferred cells were calculated as ∑[% of cells in generation (*n*) × *n*]/100 (*n* = number of generation).

### Statistical Analysis

Data are presented as mean ± SD. Statistical significance was analyzed by unpaired *t*-test or two-way ANOVA using GraphPad Prism Software. Values of *P* < 0.05 were considered to be statistically significant.

## Results

### MHC Class II Deletion on APC Impairs Expansion of CD4+ T Cells by CD28SA *In Vitro*

**Figure 1:**
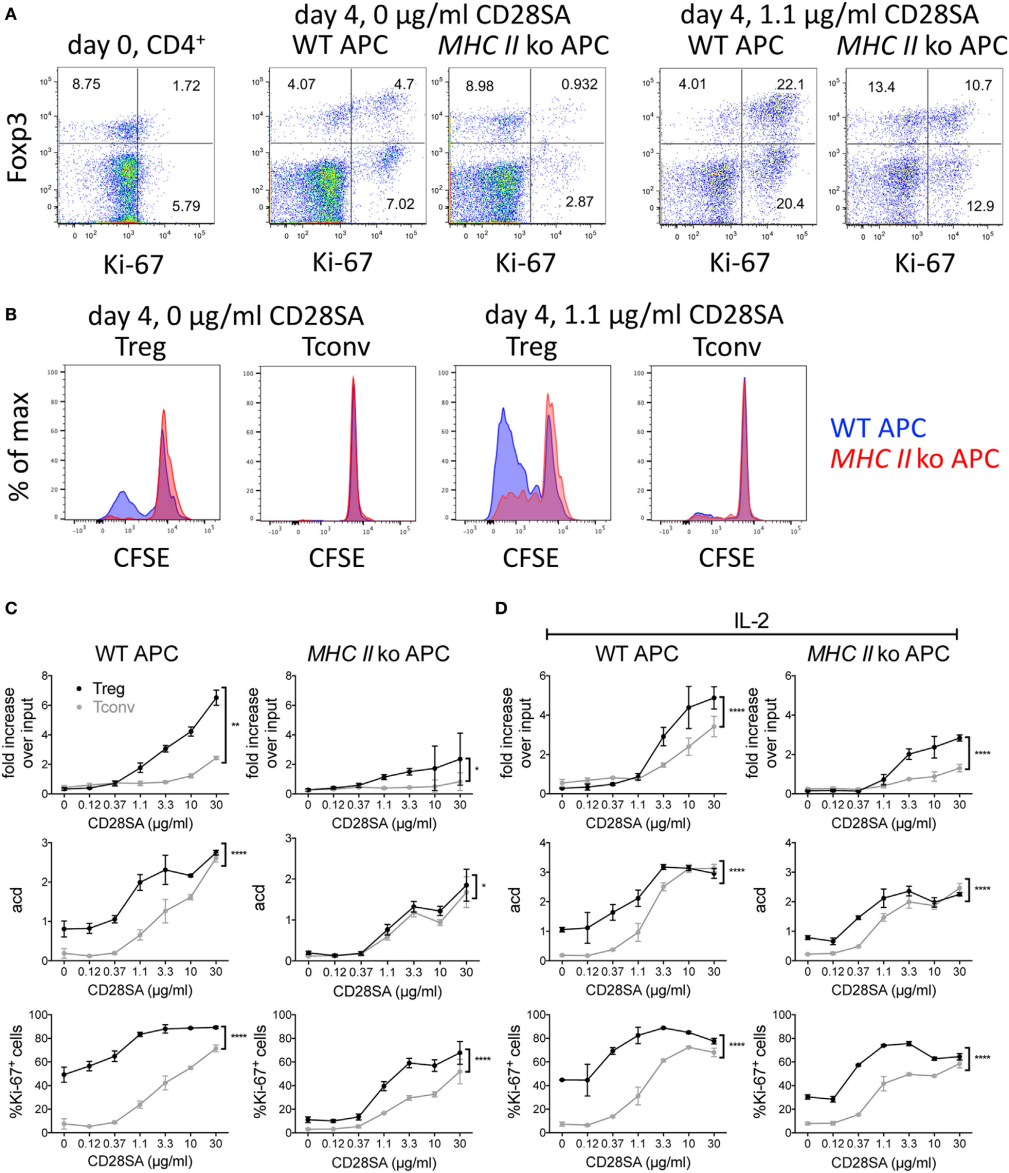
Effect of MHC II on expansion of regulatory T cells (Treg) induced by CD28 superagonists (CD28SA) *in vitro*. 1 × 10^5^ CFSE-labeled CD4^+^ T cells from C57BL/6 mice were cultured together with 2.5 × 10^5^ T cell-depleted APC of WT or *MHC II* ko mice and 0–30 µg/ml CD28SA. On day 4, cell numbers, proliferation, CD4, Foxp3, and Ki-67 expression were analyzed. **(A)** Flow cytometric analysis of Foxp3 and Ki-67 expression on day 0 and day 4 of CD4^+^ cell stimulated with WT or *MHC II* ko APCs and 0 or 1.1 µg/ml of CD28SA. **(B)** Comparison of CFSE dilution in Treg and Tconv stimulated with WT APC (blue) or *MHC II* ko APC (red). **(C,D)** Fold increase over input on day 0, average cell division (acd) and frequency of Ki-67^+^ cells of Treg (black) and Tconv (gray) in cultures with WT (left) or *MHC II* ko APC (right) in the absence **(C)** and presence **(D)** of IL-2. Fold increase over input was calculated from absolute cell numbers of Treg (CD4^+^Foxp3^+^) and Tconv (CD4^+^Foxp3^−^) in correlation with absolute cell numbers put into the culture on day 0. Average cell division (acd) numbers were calculated as ∑[% of cells in generation (*n*) × *n*]/100 (*n* = number of generation). Two-way ANOVA: *P ≤ 0.05; **P ≤ 0.01; ***P ≤ 0.001; and ****P < 0.0001. Data are mean ± SD of triplicate samples. Data are representative of at least three independent experiments with similar results.

In our initial *in vitro* experiments using mouse cells, we stimulated purified CFSE-labeled C57BL/6 CD4^+^ T cells cocultured with T cell-depleted spleen cells as APC with increasing concentrations of the mouse CD28SA D665 (5) and evaluated the number of recovered cells and of average cell divisions (acd), and expression of the nuclear proliferation marker Ki-67 in conventional and regulatory CD4^+^ T cells 4 days later. Sample dot plot and histogram data are provided in Figures 1A,B. These illustrate that even without CD28SA stimulation, coculture with MHC II^+^, but not with MHC II^−^ APC in this optimized system leads to an upregulation of Ki-67 and Foxp3 expression levels and cell division in some Treg. Inclusion of 1.1 µg/ml of D665 (“low dose”) to cultures with MHC II^+^ APC increases the frequency of Foxp3^+^ cells threefold, most of which now express Ki-67 and indeed strongly proliferate; this effect is greatly reduced if MHC II-deficient APC are employed. Tconv respond only moderately to this CD28SA dose with Ki-67 expression and CFSE dilution in only a small fraction of cells.

As shown in Figures 1C,D for CD28SA concentrations from 0.12 to 30 µg/ml, both Tconv and Treg CD4^+^ T cell numbers increased in a dose-dependent manner in the presence of WT APC. As expected, Treg expanded much more than Tconv, resulting in a sevenfold recovery over input at the highest CD28SA dose employed (30 µg/ml) as compared to a twofold recovery of conventional CD4^+^ T cells (Figure 1C). Moreover, 10-fold more CD28SA was required for Tconv than for Treg to surpass the input cell number (10 versus 1.1 µg/ml). These results were paralleled by an increase in the fraction of cells expressing the nuclear proliferation marker Ki-67 and the average number of cell divisions (Figure 1C). Furthermore, calculating the average number of cell divisions within the dividing population of each culture indicated that clonal expansion was similar for those cells that were triggered to proliferate regardless of the magnitude of the overall response (data not shown). When MHC II-deficient instead of WT APC were used, the superior clonal expansion of Treg was greatly reduced. Furthermore, the unstimulated “background” response of Treg cocultured with WT APC with regard to cell division and Ki-67 expression was lost, and the dose–response profiles of both populations became indistinguishable for Treg and Tconv cells with regard to the number of cell divisions and much more similar than in WT APC-complemented cultures with regard to Ki-67 expression. These data support the hypothesis that the preactivated status of Treg that recognize self-peptides presented by MHC II renders them more sensitive to CD28SA stimulation than Tconv cells that receive only tonic TCR signals.

In order to test whether the preferential expansion of Treg was due to IL-2 scavenging, which would inhibit proliferation of Tconv dependent on autocrine IL-2 production, exogenous IL-2 was included in parallel cultures of CD4^+^ T cells using either WT or MHC II-deficient APC (Figure 1D). While this improved the recovery and proliferative activity of Tconv somewhat, the Treg response remained superior in these parameters. Of note, the MHC II-dependent “background” responses of Treg with regard to cell division and Ki-67 expression observed in the absence of exogenous IL-2 (Figure 1C) became less MHC II dependent in the presence of IL-2, suggesting that IL-2 stimulation had maintained the Treg, which constitutively express high-affinity IL-2 receptors, in their activated state.

### Absence of an MHC II-Presented Peptide Repertoire Abolishes the Preferential Response of Treg to CD28SA *In Vivo*

**Figure 2:**
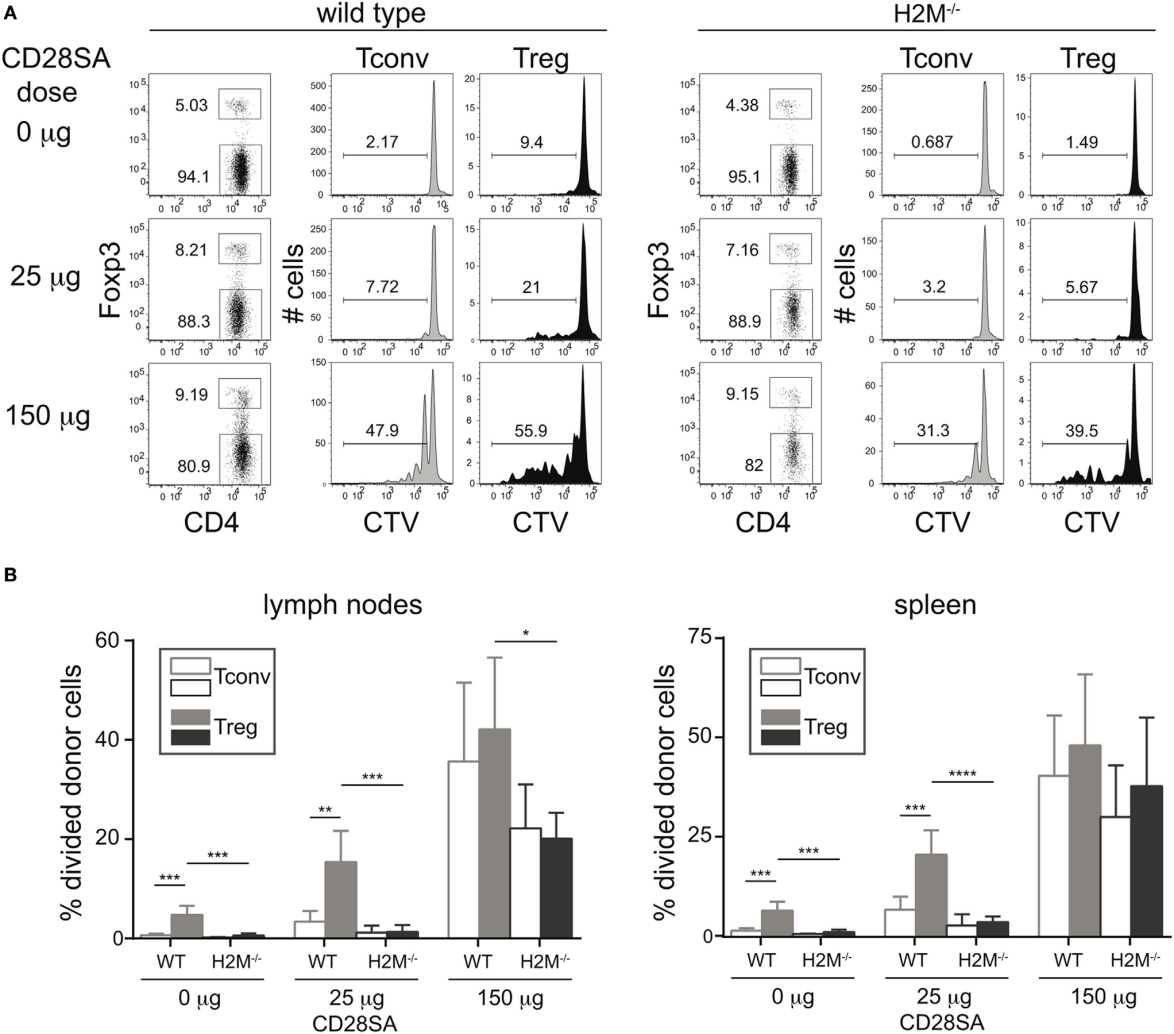
Prevention of MHC II/peptide recognition interferes with proliferation of regulatory T cells (Treg) induced by low dose CD28 superagonists (CD28SA) *in vivo*. 1 × 10^7^ Cell Trace Violet (CTV)-labeled CD4^+^ T cells from C57BL/6 mice were transferred into H2M^−/−^ or C57BL/6 mice. One day after cell transfer recipient mice received CD28SA injection (0, 25, and 150 µg). Three days later transferred cells were analyzed for proliferation, CD4 and Foxp3 expression **(A)** and frequencies of divided cells in transferred Treg (CD4^+^Foxp3^+^) and Tconv (CD4^+^Foxp3^−^) **(B)**. Unpaired Student’s *t*-test: *P < 0.05; **P ≤ 0.01; and ***P ≤ 0.001. Data are mean + SD from three mice per group. Data are representative of two independent experiments.

Since the previous experiment established the role of MHC II recognition in the CD28SA response, but failed to distinguish between the effects of self-peptide recognition from tonic TCR–MHC interactions, we addressed this issue in an *in vivo* experiment employing H-2M mutant mice. These mice have a peptide-loading defect, resulting in the expression of MHC II molecules exclusively loaded with the CLIP peptide at the cell surface (17, 19). 1 × 10^7^ CTV-labeled purified CD4^+^ T cells (Tconv and Treg) were transferred to either WT or H-2M^−/−^ mice, which were injected with 25 or 150 µg of the D665 antibody 1 day later. As shown in Figures 2A,B, high-dose application in WT recipients resulted in a similar frequency of divided Tconv and Treg, whereas low-dose stimulation resulted in a much stronger response of the Treg as compared to the Tconv subset. In H-2M knockout recipients, on the other hand, the advantage of Treg over Tconv to low-dose CD28SA stimulation was completely lost, indicating that it is indeed the recognition of self-peptides, which renders Treg highly sensitive to CD28SA-driven expansion. As expected and similar to our *in vitro* experiments, Treg also showed some spontaneous cell division in wild-type recipients, in line with their known turnover, which depends on costimulation-supported self-recognition (22, 23), but did not divide without CD28SA stimulation in H-2M^−/−^ hosts.

### Low-Dose CD28SA Application *In Vivo* Expands Treg and Partially Inhibits Expansion of Conventional Autoreactive T Cells

**Figure 3:**
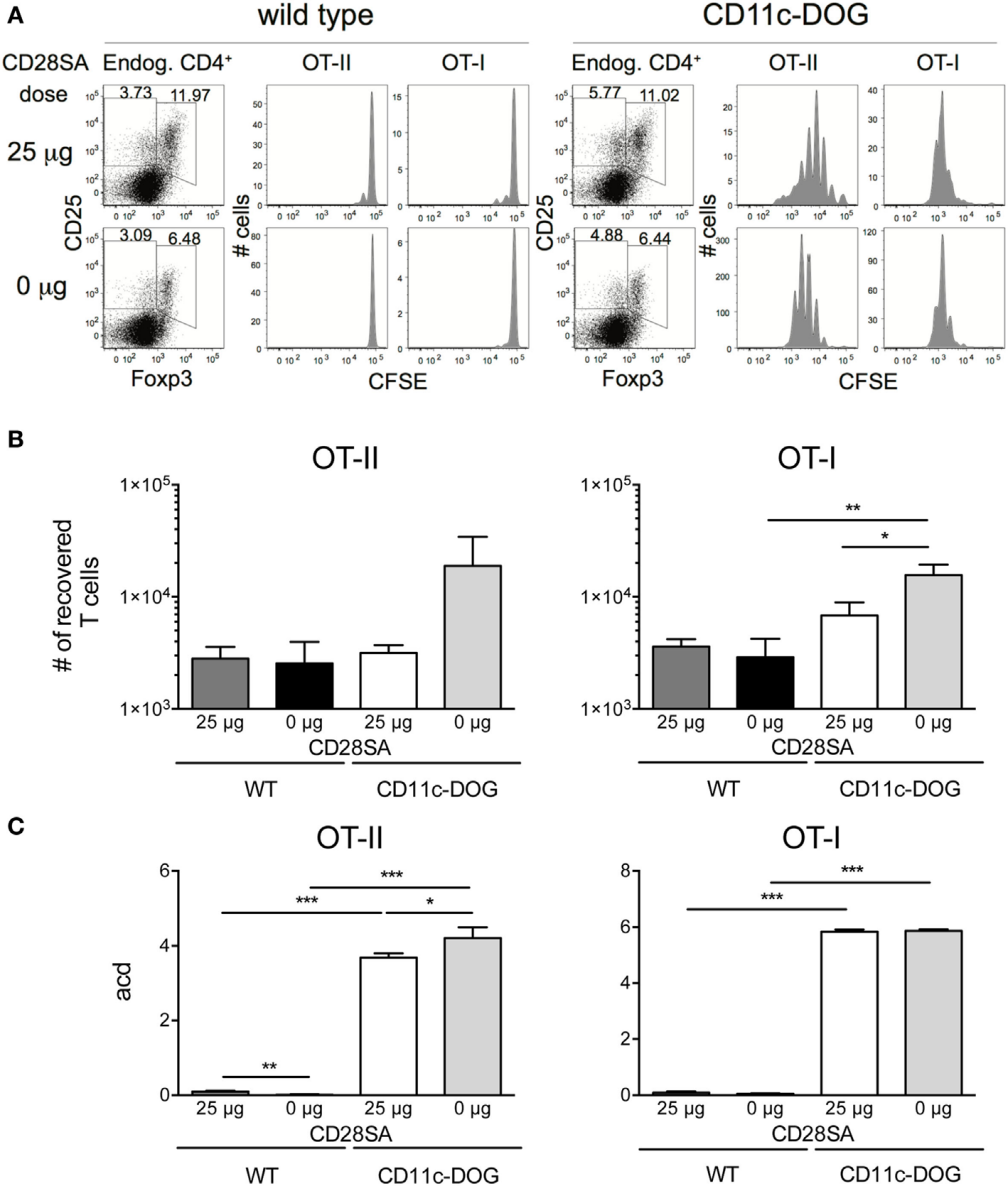
Low-dose stimulation with CD28 superagonists (CD28SA) reduces expansion of conventional autoreactive T cells *in vivo*. 3 × 10^4^ CD8^+^Thy1.1^+^ T cells from OT-I Thy1.1^+/−^ mice and 6 × 10^4^ CD4^+^Thy1.1^+^ T cells from OT-II Thy1.1^+/+^ mice were CFSE-labeled and transferred into congenic CD11c-DOG or C57BL/6 mice. 12 h later recipient mice received CD28SA injection (0 or 25 µg). **(A)** Three days after cell transfer endogenous cells in lymph nodes were analyzed for CD4 and Foxp3 expression and transferred CD4^+^ and CD8^+^ cells for CFSE dilution. Data represent absolute cell numbers in transferred T cell **(B)** and numbers of average cell divisions (acd) **(C)**. Average cell division (acd) numbers were calculated as ∑[% of cells in generation (*n*) × *n*]/100 (*n* = number of generation). Unpaired Student’s *t*-test: *P ≤ 0.05; **P ≤ 0.01; ***P ≤ 0.001; and ****P < 0.0001. Data are mean + SD from three mice per group. Results are representative of two independent experiments.

Since autoreactivity of Treg is important for their preferential response to low-dose CD28SA, it has to be considered that autoreactive and thus potentially pathogenic conventional T cells might also be further expanded under these conditions, thereby abrogating the protective effect of CD28SA therapy in autoimmunity. While in all of the many rodent models of autoimmunity studied (24), decreased rather than increased autoimmune pathology has been observed as a result of CD28SA treatment, we nevertheless directly tested this possibility by injecting 3 × 10^4^ CFSE-labeled OVA/K^b^-specific OT-I and 6 × 10^4^ OVA-IA^b^-specific OT-II cells into CD11c-DOG mice which express OVA in dendritic cells (20) or, as a control, into WT C57BL/6 mice. 25 µg of CD28SA was injected 12 h later in order to allow the initial activation of the autoreactive conventional CD8^+^ and CD4^+^ T cells. Three days later, the number and proliferative history of the recovered cells were determined along with the effect of CD28SA stimulation on endogenous Treg. As seen in Figure 3A, OT-I and OT-II cells both divided extensively in CD11c-DOG, but not in control recipient mice. Moreover, CD28SA treatment resulted in the expected expansion of host Treg in both types of recipients. Importantly, the number of OT-I and OT-II cells recovered from lymph nodes from CD11c-DOG mice was not increased but rather decreased by CD28SA treatment (Figure 3B, log scale). For OT-II cells, the number of cell divisions induced by *in vivo* recognition of OVA was also reduced by this treatment (Figure 3C), whereas an effect on the proliferative history of OT-I cells was not discernable, presumably because the ability to detect cell division by this assay was in saturation. Note that the cells harvested from lymph nodes do not reflect the total number of cells generated in response to the self-antigen OVA in the mouse and that both expansion and cell death contribute to the final score. Hence, differences in acd are not expected to directly correspond to the differences in the number of OT-I and OT-II cells recovered from lymph nodes. Nevertheless, these data collectively show that the expansion of autoreactive cells within the conventional CD4^+^ and CD8^+^ T cell populations is negatively influenced in CD28SA-treated mice, most likely because they are controlled by the much larger population of concomitantly activated Treg.

### *In Vitro* Blockade of HLA II Interferes with the Preferential Expansion of Treg in Human PBMC Cultures

**Figure 4:**
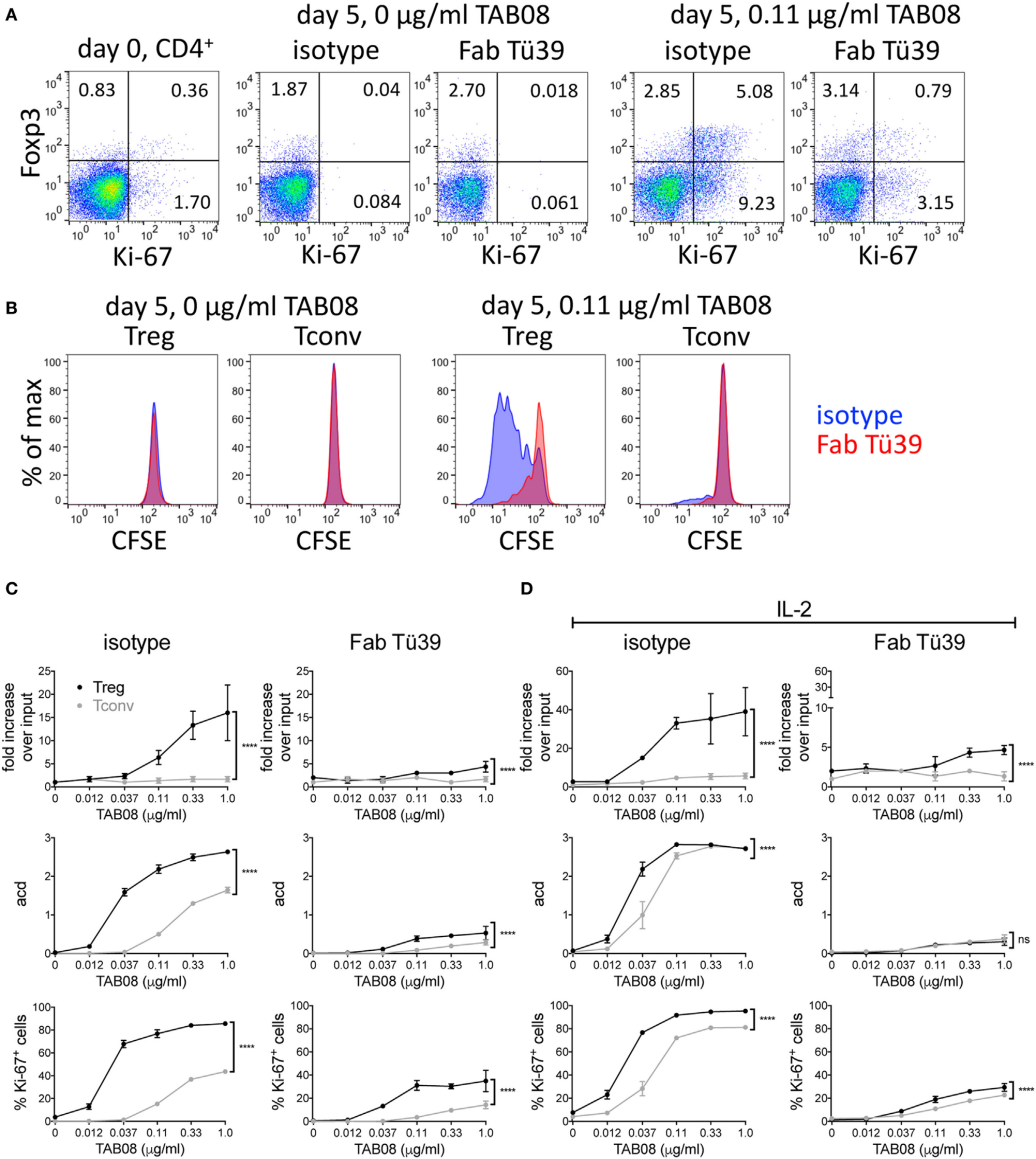
HLA II blockade interferes with TGN1412-induced expansion and proliferation of human regulatory T cells (Treg). High-density precultured peripheral blood mononuclear cells (PBMC) were cultured in 0.6 ml AB medium for 5 days at 1 × 10^6^ cells/ml in 48-well flat-bottom tissue culture plates. PBMC were stimulated with TAB08 in the presence or absence of 10 µg/ml of the Fab fragment of the pan-HLA II-reactive monoclonal antibodies (mAb) Tü39 (Fab Tü39) or a mouse IgG2a isotype control mAb. **(A)** Flow cytometric analysis of Foxp3 and Ki-67 expression on day 0 and day 5 of stimulation with 0.11 µg/ml TAB08 in the absence or presence of HLA II blockade. **(B)** Comparison of CFSE dilution in Treg and Tconv stimulated with 0.11 µg/ml TAB08 in the absence or presence of HLA II blockade. **(C,D)** Fold increase over input on day 0, average cell division (acd), and frequency of Ki-67^+^ cells of TAB08-stimulated Treg (black) and Tconv (gray) in the absence (left) or presence (right) of HLA II blockade without **(C)** or with **(D)** addition of IL-2. Fold increase over input was calculated from absolute cell numbers of Treg (CD4^+^Foxp3^+^) and Tconv (CD4^+^Foxp3^−^) in correlation with absolute cell numbers put into the culture on day 0. Average cell division (acd) numbers were calculated as ∑[% of cells in generation (*n*) × *n*]/100 (*n* = number of generation). Two-way ANOVA: ****P < 0.0001; ns = not significant. Data are mean ± SD of triplicate samples. Data are representative of at least three independent experiments with similar results.

Freshly isolated human PBMC fail to respond to the human CD28SA TGN1412/TAB08 in soluble form because they have lost the tonic signals generated by T cells in the tissues through cellular interactions (9). We have recently introduced a system, which corrects this defect (9). In brief, PBMC, which are refractory to TAB08 stimulation when freshly isolated, are precultured at high cell density for 2 days to allow cellular interactions and thereby restoration of tonic signaling in the T cells, rendering them highly reactive to CD28SA stimulation. Such “RESTORE” cultured PBMC were CFSE labeled and stimulated for 5 days with graded concentrations of TAB08, and the role of MHC II recognition was tested by blockade with Fab fragments derived from the pan-HLA II-reactive mAb Tü39. Note that the stimulatory activity of TAB08 for human T cells is about 10-fold higher than that of D665 for mouse cells (compare Figures 1 and 4). Panels A and B of Figure 4 show representative dot plots and histograms using 0.11 µg/ml TAB08 for stimulation. Complete titrations are shown in Figure 4C, and in Figure 4D for IL-2 supplemented cultures to account for possible IL-2 scavenging effects of Treg cells on Tconv proliferation.

CD28 superagonist stimulation resulted in a much larger increase in recovered Foxp3^+^ as compared to Foxp3^−^CD4^+^ T cells both in the absence (Figure 4C) and in the presence of exogenous IL-2 (Figure 4D). This is in agreement with our recently published results, where we also demonstrated that CD28SA expanded CD4^+^Foxp3^+^ cells are functional Treg (13). While the extent of this increase (up to 40-fold) is not sufficiently explained by the number of cell divisions but is, in part, due to downregulated Foxp3 expression in unstimulated PBMC (unpublished observations, see Discussion), the increase in the average number of cell divisions and the frequency of Ki-67 expressing cells independently show the superior sensitivity of Treg to CD28SA stimulation.

To investigate the role of HLA II recognition in this phenomenon, we included Fab fragments derived from the pan-HLA II-reactive mAb Tü39 in parallel cultures. This strongly reduced the proliferation and cell recovery of both subsets and abolished the superior reactivity of Treg to very low CD28SA doses. Thus, also human Treg rely on MHC II recognition for their high sensitivity to CD28SA stimulation, presumably because they receive full stimulation through the recognition of self-peptide/HLA II complexes.

## Discussion

In view of the still increasing list of disorders that have been shown in rodent models to respond to Treg-based therapies, which have moved beyond immunopathologies to include dysregulated and damaged tissues, e.g., after heart attack, myocardial infarction, inflammation of adipose fatty tissue, and muscle injury (25–27), the strategy of inducing a transient wave of polyclonal activation of Treg that seek out inflamed and damaged sites seems to be attractive. Beyond the presently studied approach of CD28SA-mediated induction of Treg proliferation, IL-2/anti-IL-2 complexes with selective binding to high-affinity IL-2R have shown such Treg-promoting activity in mice (28), and a CD4-specific mAb binding to a specific epitope on human CD4^+^ T cells was reported to have a similar effect (29).

With regard to CD28SA-mediated polyclonal Treg activation, pronounced therapeutic effects have been observed not only in rodent models for major human autoimmune diseases such as rheumatoid arthritis, multiple sclerosis, or type 1 diabetes (14, 30, 31) but also in graft versus host disease (32, 33), solid-organ transplantation (34–36), infection-associated inflammation (37), and recovery from myocardial infarction (25) and stroke (38). Moreover, CD28SA-activated Treg were indeed shown to switch to a tissue-migrating and IL-10-secreting phenotype and to accumulate at sites of insult (39).

While the original description of the preferential response of Treg to CD28SA in rats was more than a decade ago (40), before being confirmed in mice (15) and, most recently, in humans (13), the mechanistic basis for this effect had not been addressed so far. In fact, the disastrous 2006 FIH study with the human CD28SA TGN1412 (now called TAB08) (11), which resulted in a life-threatening release of pro-inflammatory cytokines from CD4EM cells, obviously questioned the concept of a particular sensitivity of Treg over T-effector cells to CD28SA stimulation. In the meantime, it is clear that at the dose applied during this study, maximum activation of both Treg and T effectors occurred (13). This had gone unnoticed in preclinical toxicity studies because CD4EM cells of humans, but not of the macaques employed, express CD28 (41), and human PBMC fail to respond to soluble CD28SA *in vitro* for lack of a preactivated signaling machinery which is switched off upon exit from tissue into the blood stream (9). Indeed, just as had been reported for rats (14) almost 10 years earlier, dose-reduction suffices to restrict the human CD28SA response to Treg (13).

Here, we tested the hypothesis that it is the autoreactivity of Treg itself which makes them so particularly sensitive to CD28SA-mediated stimulation. This idea was based on the observation that in contrast to what was originally thought, CD28SA stimulation is strictly dependent on the presence of a functional TCR signaling machinery (7, 8), which provides the SLP76/Itk signalosome as a substrate for amplification. However, because of the strong CD28 signaling input at a high level of crosslinking of CD28 by CD28SA, weak “tonic” TCR signals suffice as a substrate for signal amplification. Accordingly, we hypothesized that the stronger TCR signaling input achieved by self-recognition of Treg would be enough to activate Treg at a lower level of CD28SA stimulation. Indeed, both murine (42, 43) and human Treg (44) constantly turn over *in vivo*. That this is driven by a costimulated response to self-peptides is indicated by the higher level of TCRζ phosphorylation in Treg as compared to Tconv (16) and by a massive reduction of this turnover upon induced deletion of CD28 in Treg (23).

Here, we show that prevention of MHC II recognition *in vitro* (mice, humans) interferes with the Treg and Tconv CD4^+^ T cell responses to CD28SA, in particular at low doses where the stronger excitability of Treg as compared to Tconv is most obvious (Figures 1 and 4).

With regard to human PBMC, the fold Treg expansion we presently report (up to 40-fold in 5 days) is surprising and cannot be explained by the number of cell divisions (three, resulting in eightfold expansion). This discrepancy is explained by a low level of Foxp3 expression in a large fraction of circulating T cells, which is caused by transient cytokine withdrawal (45), leading to a failure to detect a large fraction of them as CD4^+^Foxp3^+^ cells. Cytokine, TCR, or CD28SA stimulation leads to rapid upregulation of Foxp3 in these “latent” Treg (compare Foxp3 levels on day 0 and day 4, Figure 4A).

Beyond a requirement for MHC II expression in the CD28SA response as such, we directly tested the hypothesis that the recognition of self-peptides is of key importance for the high sensitivity of Treg to CD28SA stimulation. This was achieved by transferring a mixture of CFSE-labeled Tconv and Treg into H-2M knockout mice which express MHC II loaded only with the CLIP peptide (17, 19). In this situation, which allows tonic but not cognate TCR signaling to occur, the ability of Treg to respond to CD28SA at a low dose which is unable to trigger Tconv was greatly diminished (Figure 2). Moreover, when a high dose of CD28SA was applied which was known to trigger proliferation of both types of CD4^+^ T cells, both responded with proliferation in the same fashion, suggesting that in H-2M knockout hosts, they both relied on tonic TCR signals for their activation by a strong CD28 signal.

While our present results clearly identify cognate self-recognition by Treg as a prerequisite for their high sensitivity to low-dose CD28SA stimulation, other cell-intrinsic differences between signaling cascades in Treg versus Tconv [reviewed in Ref. (46)] as well as the ability of Treg to scavenge IL-2 (47) are likely to additionally contribute to the Treg-dominated expansion at low CD28SA doses.

At face value, our present results suggest that low-dose CD28SA treatment might not only activate Treg but also autoreactive Tconv, thereby exacerbating autoimmunity. This has, however, not been observed in any of the multiple models of autoimmune diseases investigated so far. We now hypothesized that the activation of, potentially, all Treg would keep a small population (the autoreactive part) of Tconv effectively under control, even if they are themselves responsive to low-dose CD28SA stimulation because of their preactivation by self-recognition. Indeed, low-dose CD28SA treatment decreased rather than increased the expansion of both CD4^+^ and CD8^+^ TCR-transgenic Tconv in hosts expressing their cognate antigen, where they strongly expand without further CD28SA treatment (Figure 3). Thus, in addition to being preferentially activated by CD28SA treatment at the population level, CD28SA-activated Treg efficiently dampen responses of autoimmune T cell clones within the Tconv population.

Importantly, our conclusion from mouse experiments that the preferential response of Treg to low-dose CD28SA stimulation depends on MHC II recognition was reproduced *in vitro* using human PBMC that had been reset to tissue-like conditions to allow a CD28SA response (Figure 4). After the promising results of low-dose application of the human CD28SA TGN1412/TAB08 to humans, which resulted in selective release of the Treg signature cytokine IL-10 into the bloodstream (13), these mechanistic insights should lend further support to the development of CD28SA-based Treg activation in a wide spectrum of human pathologies in which, according to the corresponding rodent models, Treg may contain or even heal the disease.

## Ethics Statement

All animal experiments were performed according to the relevant US and German regulations for animal experimentation and approved by local authorities (Institutional Animal Care and Use Committees at the La Jolla Institute for Allergy and Immunology and Regierung von Unterfranken).

## Author Contributions

DL, PT, and TH designed the experiments. DL, PT, TG, BB, CS, NB, and SB contributed to performing and analyzing the experiments and interpreting the results. TH directed the study and wrote the manuscript, with input from DL and PT.

## Conflict of Interest Statement

TH is a consultant to TheraMAB LLC, the company developing CD28SA therapy. All other authors declare that the research was conducted in the absence of any commercial or financial relationships that could be construed as a potential conflict of interest.

## Abbreviations

CD4EM, CD4 effector memory; FIH, first-in-human; Treg, regulatory T cells; Tconv, conventional CD4^+^ T cells; CTV, cell trace violet; CD28SA, CD28 superagonists.
